# Research and Implementation of Text Generation Based on Text Augmentation and Knowledge Understanding

**DOI:** 10.1155/2022/2988639

**Published:** 2022-09-10

**Authors:** Lei Liu, Yeguo Sun, Yihong Liu, Rachel Edita O. Roxas, Rodolfo C. Raga

**Affiliations:** ^1^School of Computer Science, Huainan Normal University, Huainan, China; ^2^School of Computing and Information Technologies, National University, Manila, Philippines; ^3^School of Finance and Mathematics, Huainan Normal University, Huainan, China

## Abstract

Text generation has always been limited by the lack of corpus data required for language model (LM) training and the low quality of the generated text. Researchers have proposed some solutions, but these solutions are often complex and will greatly increase the consumption of computing resources. Referring to the current main solutions, this paper proposes a lightweight language model (EDA-BoB) based on text augmentation technology and knowledge understanding mechanism. Experiments show that the EDA-BoB model cannot only expand the scale of the training data set but also ensure the data quality at the cost of consuming little computing resources. Moreover, our model is shown to combine the contextual semantics of sentences to generate rich and accurate texts.

## 1. Introduction

**Figure 1:**
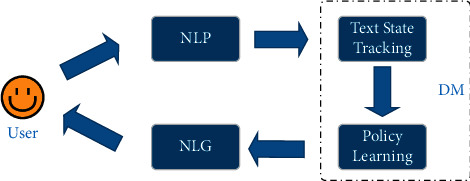
NLG framework.

Natural language generation (NLG) is an essential and challenging task in the current natural language processing (NLP) field [1]. And text generation task usually takes text as input, uses the word vector to represent it semantically, and finally, generates understandable natural language text. In Figure 1, all traditional NLP tasks can be transformed into text generation tasks. Typical text generation tasks include machine translation, document summarization, and dialogue systems. Since the Seq2Seq [2] framework was proposed in 2014, text generation has rapidly become a hot research topic. The Seq2Seq is based on an encoder-decoder mechanism, in which the encoder maps input text sequences to fixed-size vectors, and the decoder maps the vectors to target sequences. This new scheme solves the problem of many-to-many forms in NLP tasks and the varying length of input and output sequences by adding a semantic encoding layer. With the recent resurgence of deep learning technologies, the deep neural NLG model has remarkably enabled machines to understand and generate natural language [3]. And researchers have proposed a series of classic and practical models, such as recurrent neural network (RNN), convolutional neural network (CNN), and transformer [4]. Based on these models, the attention mechanism and the copy/pointer-generator [5] have also extensively promoted the research of text generation.

But the current development of text generation also faces some problems [6]. For instance, the quality of generated text is often limited due to the lack of large-scale and high-quality annotated corpus data for deep neural NLG model training. In addition, due to the limited knowledge contained in the input text, LM generally suffers from inability to understand language well, retain and recall knowledge using memory, and reason about complex concepts and relational paths. These problems often limit the quality of the output text by the NLG model.

**Figure 2:**
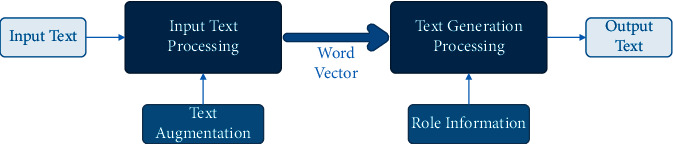
Our scheme framework (1).

Consequently, this paper intends to use text augmentation to generate expanded training corpus data. And, we will use a solution based on a knowledge understanding mechanism to solve the second problem. The framework of our scheme is shown in Figure 2.

The primary advantages of the scheme are summarized as follows:

1. We solve the problem of limiting language model training due to the lack of corpus data by introducing lightweight text enhancement technology and ensuring training data quality.
2. Our scheme adopts a multi-LM combination scheme, which meets the model's needs for both knowledge understanding and text generation tasks.
3. We successfully combined the abovementioned two parts of the work. Based on this, this paper improves the performance of text-generating language models on a limited-scale corpus.
4. This paper designs a simplified training data processing method and proposes a scheme to reasonably evaluate the generated text with a few indicators. These methods are beneficial to the realization and evaluation of text generation schemes.

In general, our model is a lightweight language model. And the EDA data augmentation method can greatly expand the training corpus data with a simple method and less resource consumption, which will help improve the accuracy of the language model. In addition, the knowledge understanding mechanism introduced by the model can combine the context information of sentences to generate richer and more accurate texts. This lightweight model makes it possible to obtain high-quality text generation in limited resource scenarios.

The remainder of our paper is organized as follows: Section 2 briefly explains the related work. Our scheme is detailed in Section 3. We discuss the performance evaluation results in Section 4. Finally, Section 5 is the conclusion.

## 2. Related Work

In this section, we introduce the current trend of text augmentation and text generation in detail. And, we compare the traditional scheme and the deep learning-based scheme of text augmentation. Meanwhile, we enumerate several general methods for text generation, and the method based on the knowledge understanding mechanism will be introduced emphatically.

### 2.1. Text Augmentation

With the development of AI, the requirements for the scale and quality of data for neural network models are gradually increasing. If the amount of data in different categories is very different in classification tasks, the model will overfit, which will seriously affect prediction accuracy. In this regard, data augmentation is a powerful solution. By using limited labeled data, more training data can be obtained. The phenomenon of overfitting in the network can be reduced, thereby training a model with more vital generalization ability.

Data augmentation techniques were initially applied in computer vision, mainly using various techniques to generate new training samples [7], which can create new data by translating, rotating, compressing, and adjusting the colors of images. Although the “new” sample data changes its appearance to a certain extent, the labels of the sample data always remain unchanged, which is beneficial to ensure the accuracy of the sample data. Consequently, data augmentation is a simple and cost-effective solution for augmenting training sample data. But the sample data in NLP is discrete, making it impossible to achieve data augmentation by simply transforming the input data because replacing one word may change the meaning of the entire sentence [8]. Accordingly, this paper presents traditional and deep learning methods for text enhancement schemes, respectively.

### 2.2. Traditional Text Augmentation Method

The traditional data augmentation method in the NLP field can be mainly divided into two types. The first one is the back-translation method, and the other one is the noise-addition method, both of which are supervised methods [9].

The back-translation method translates the original data into other languages and then translates it back to the original language [10]. This method is a data augmentation technique frequently used by NLP in machine translation and has been successfully used in several malicious comment classification competitions [11]. The language logic order of back-translation methods is different, which can increase the diversity of text data. Therefore, this method not only change the syntactic structure but also preserve the semantic information, which can generate new data, that is, very different from the original data. However, the data produced by back-translation methods are often too dependent on the quality of the translation system and may not be accurate in most cases. If the interface of some translation software is used, the user may also encounter situations such as account restrictions.

**Figure 3:**
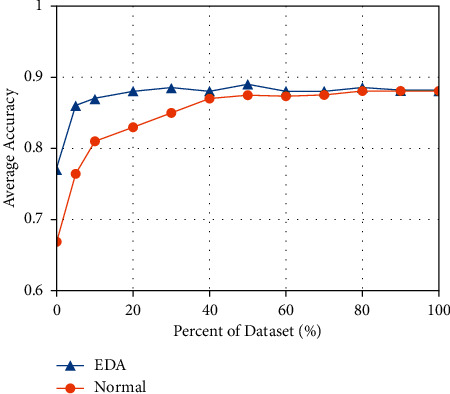
EDA performance comparison.

Correspondingly, the noise-addition method is to create new data similar to the original data by replacing words, deleting words, etc., on the basis of the original data [12]. Some papers use Gaussian noise, dropout noise, etc., to augment data. Nevertheless, the methods represented by easy data augmentation for text classification tasks (EDA) [13] use simpler algorithms to implement data augmentation in a combined form. And the EDA is the first method to explore text editing techniques for data augmentation comprehensively. By systematically evaluating the performance of EDA on multiple benchmark classification tasks, it is shown that EDA can provide substantial improvements to the training data of the model, especially on smaller datasets, as shown in Figure 3.

EDA mainly includes four methods: synonyms replace (SR), randomly insert (RI), randomly swap (RS), and randomly delete (RD). Reasonable use of these four methods can quickly and easily expand the scale of training samples, but there are also disadvantages. For instance, RI may cause the original training data to lose semantic structure and order. The addition of synonyms does not focus on the keywords in the sentence, and the diversity of data expansion will be more limited. On the other hand, RS does not change the morphemes of the original sentence, so the generalization ability of new sentence patterns, sentence patterns, and similar words is substantially improved. In practical research, the combination of SR and RS is often used.

### 2.3. Text Augmentation Method Based on Deep Learning

With the widespread application of machine learning, researchers have also done a lot on deep learning-based text augmentation methods. These text augmentation schemes can mainly be divided into semi-supervised learning and unsupervised learning methods. The semi-supervised learning method is proposed to use unlabeled data better and reduce the model's dependence on large-scale labeled datasets [14, 15]. Experiments have shown that this is a powerful learning paradigm. MixMatch [16] proposed by Google represents the semi-supervised learning method, which incorporates consistency regulation, pseudo label idea, entropy regularization, and MixUp technology. It works by guessing the low-entropy labels of unlabeled samples produced by the MixUp data augmentation method and mixing the unlabeled data with the labeled data. These methods enable MixMatch to achieve significantly better results than previous semi-supervised learning techniques.

Unsupervised data augmentation (UDA) is also a successful data augmentation method [17]. Its success benefits from the adoption of mechanisms that use specific goals for specific tasks, that is, the targeted data augmentation (TDA). Compared to regular noise, this task-specific mechanism can generate more diverse and realistic noise and learn how to find missing or most wanted training signals in the original labeled dataset.

For unlabeled data, UDA uses KL divergence for augmented unlabeled data prediction results, which is different from MixMatch. The specific target data enhancement of TDA includes three methods: back translation, AutoAugment, and TFIDF. The Back Translation method can help enrich the sentence patterns and patterns of the data, and the TFIDF method optimizes UDA's random word processing strategy. In addition, another critical breakthrough of UDA is the use of the training signal annealing (TSA) method to gradually release the training signal during training, which avoids the situation that the labeled data and unlabeled data may be very different.

In general, traditional methods perform better on small batches of data. While deep learning-based methods can undoubtedly meet the needs of large-scale data, they are expensive to implement relative to the performance gain, so they are not often used in practice.

### 2.4. Text Generation

**Figure 4:**
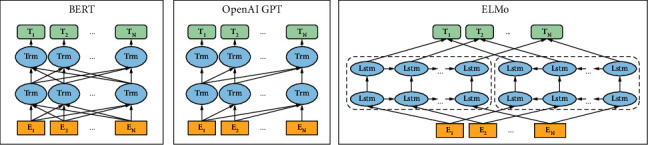
Structural comparison of Bert, GPT, and ELMo.

The text generation method is the foundation of many NLP tasks [18–20]. And the general text generation model is mainly based on the encoder-decoder framework [21]. The encoder learns to encode the input text into a vector representation, and the decoder is responsible for decoding this vector representation into a text sequence. In recent years, the blessing of technologies such as neural networks and deep learning has extensively promoted the development of text generation [22]. Since the Seq2Seq [23] model was proposed in 2014, text generation has rapidly become an important research point, and researchers have launched a series of classic and practical language models. The pretraining model has become a research trend in the current NLP field, and of course, they are also widely used in text generation. Its general and flexible framework design features can effectively solve the problem that the quality of the model is affected by the lack of labeled training data and provide good support for downstream tasks through fine-tuning. Based on these models, the attention mechanism and copy/pointer-generator mechanism has also greatly promoted text generation research [24]. Typical pretraining models include ELMo [25], GPT [26], and Bert [27] as representatives. Because Bert has added transformer [28] and bidirection technologies based on ELMo and GPT, it has become the most popular NLP language model.  Figure 4 is a comparison frame diagram of Bert, GPT, and ELMo.

**Figure 5:**
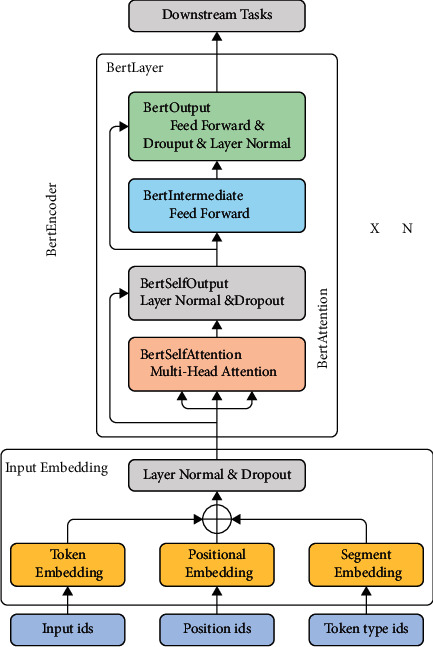
Bert network.

As a general model of NLP, Bert has been widely used in various scenarios, and its multi-stacked transformer network structure based on bidirectional encoding can significantly improve the expressiveness of the model. Meanwhile, Bert added MLM and NSP tasks in the pretraining process, making the trained model have the representational solid ability.  Figure 5 shows the chart of Bert's network framework.

**Figure 6:**
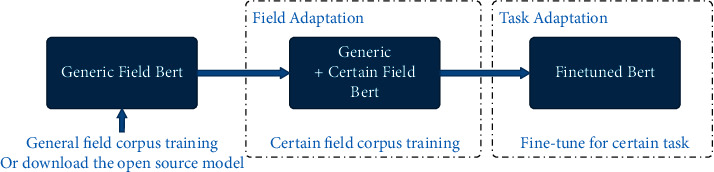
Bert's task processing flow.

Meanwhile, Bert can provide significant support for various downstream tasks. Especially, when dealing with classification tasks, sequence labeling tasks, and knowledge reasoning tasks, you only need to add the relevant structure of the corresponding task after Bert's output without adjusting Bert. However, Bert is not naturally suitable for text generation tasks due to the inconsistency between Bert's MLM mechanism and the goal of generating tasks. It can be solved by adjusting Bert's mask matrix. The specific way is to make Bert predict the current word only based on the previous content of the text and ignore the latter content to continuously generate the word at the current position until the predicted word is (CLS) label, which is similar to the principle of the UNILM [27]. As shown in Figure 6, Bert can almost be competent for all NLP downstream tasks after field adaptation and task adaptation.

However, researchers also found that traditional text generation methods only rely on input text to generate, lacking richer “knowledge” information, so the generated text is often boring and lacks interesting content [29]. For text generation tasks, knowledge can transcend the semantic limitations in the input text and help the text generation system to generate richer and more interesting text. Therefore, knowledge-enhanced text generation has become the focus of researchers.

In the text generation task, knowledge can be obtained through different methods and information sources, including keywords, topics, language features, knowledge bases, knowledge maps, and the acquisition technology is very mature. Based on this, researchers have widely incorporated knowledge into text generation models to improve the quality of text generation. Reference [30] introduces an attention mechanism, which is mainly used to describe the importance of the input text to the generation process by adding a context vector to the decoder. Reference [31] proposed the CopyNet framework, which designed knowledge patterns and knowledge-related dictionaries for knowledge and used copy/ pointer-generator mechanism for the generation of text output sequences. Reference [32] proposes an approach to memory networks, a recurrent attention model that acts on external storage by cyclically exploiting the input sequence to read the memory representation on the storage, and then write the updated memory representation back to the storage superior. The pretraining model proposed in [33] is currently a widely used solution. By using large-scale unlabeled datasets for pretraining models, these models can provide better model initialization for the text generation task model to solve the problem of insufficient generalization ability. The proposed methods not only increase the sentiment of the generated text but also effectively combine the context and contextual information to improve the accuracy of text generation.

## 3. Our Scheme

**Figure 7:**
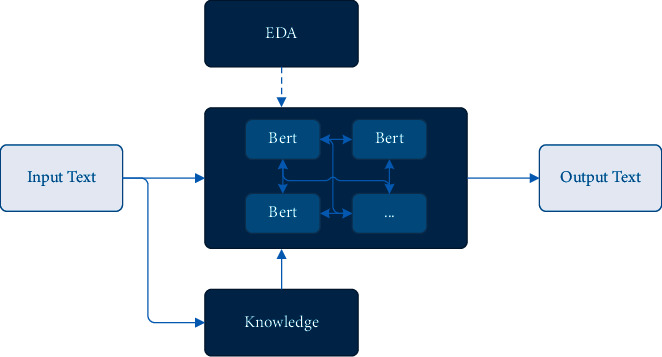
Our scheme framework (2).

As mentioned above, EDA can achieve better text augmentation effects in small-scale data application scenarios. The improved pretrained language model (Bert) can meet the needs of text generation after introducing the knowledge understanding mechanism. Therefore, our scheme will be based on EDA and Bert, as shown in Figure 7. First, the high-quality annotated training sample data of the text generation model is boosted by EDA; second, the multi-Bert combination scheme is adopted to solve the knowledge understanding task and the text generation task simultaneously.

### 3.1. Our EDA Method

The main body of EDA is four data augmentation algorithms, SR, IR, RS, and RD. And the SR and RS are suitable for short texts, while the RI and RD are more suitable for text augmentation of long texts. In addition, it also includes important configuration parameters such as *n* and *α*. Among them, we use *n* to control the number of target sentences generated corresponding to each source sentence, and *α* is a parameter that indicates the percent of the words in a sentence is changed by augmentation. Since the change of the words in the sentence may change the semantics of the sentence, many factors need to be comprehensively considered when selecting the four algorithms of EDA and setting *n* and *α*.

**Figure 8:**
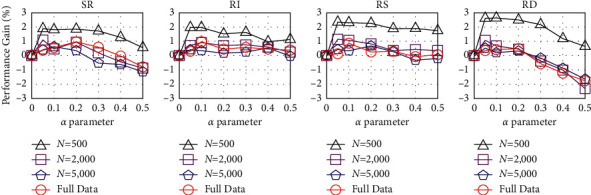
Average performance gain of EDA.

**Figure 9:**
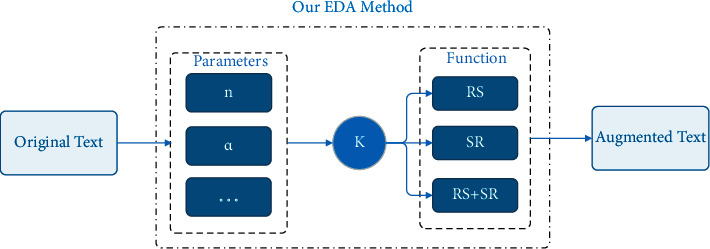
Our EDA method is based on SR and RS.

Figure 8 shows that the average performance gain of EDA operations over five text classification tasks for different training set sizes, while varying the augmentation parameter *α* = {0.05, 0.1, 0.2, 0.3, 0.4, 0.5}. From the results, the average performance of the four algorithms of EDA is different. In contrast, the gain of RI on performance is relatively low, RD is greatly affected by the change of *α* value, and the realization effect of SR and RS is relatively good. Since the language model in this paper is mainly oriented to short texts, to reduce the impact of RI and RD on the semantics of the original text as much as possible, this paper only uses the SR and RS algorithms based on setting the *α* value at a low value. In Figure 9, our new EDA method uses random operator *K* to control our method to choose whether to use SR, RS, or SR + RS. We mainly use SR or RS for short texts. For longer texts, we use the SR + RS algorithm more. In the SR + RS algorithm, *α* is divided into *α*1 and *α*2, respectively, used to control the number of words in the text for synonym replacement and random swap.

**Figure 10:**
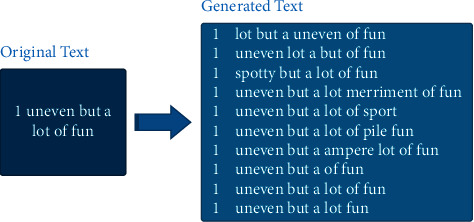
Text augmentation results.

Figure 10 shows the augmentation result of one sample text when *n* = 10 and *α* = 0.1.

### 3.2. Text Generation Model (EDA-BoB)

On the one hand, the problem of inconsistency in dialogue responses is one of the biggest challenges in the NLP field, and it is difficult to eradicate this phenomenon with large-scale training data alone. The language model based on knowledge understanding can better understand the contextual information to generate richer and more accurate text. Language models can understand many kinds of knowledge, and the user role information is widely used because of its simple structure and easy training. On the other hand, since the independent Bert can only handle a specific NLP task, to enable the language model to handle both the knowledge understanding task and the text generation task, we adopt the Bert-over-Bert (BoB) model proposed in [34]. BoB is a new model based on the combination of multi-Bert, including a Bert encoder and two Bert decoders, in order to separate the acquisition of understanding ability and generation ability. Once, we separate the two, whether character information understanding or text generation, we can find sufficient data resources for training.

**Figure 11:**
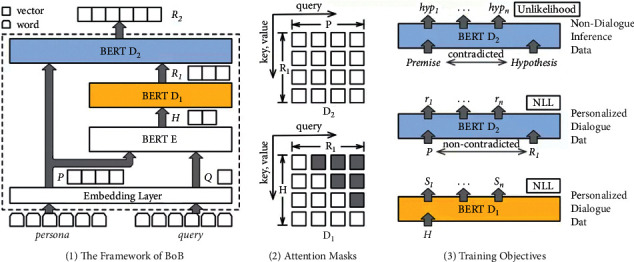
BoB framework and training objectives.

The model consists of an encoder *E*, an autoregressive decoder *D1* for responding to dialogue replies, and a bidirectional decoder *D2* for consensus understanding. Given role information *P* and dialogue input *Q*, *E*, and *D1*, the model operates in a classical encoder-decoder mode to learn a typical input-to-reply mapping *F*_*G*_*(S|Q, P)* and generate a preliminary dialogue-reply representation *R1*. Then *R1* and persona *P* are fed to a bidirectional decoder *D2* to map *R1* to the final response representation *R2: F*_*U*_*(R2|S, P)*. Since the part *F*_*U*_*(R|S, P)* that learns consistent understanding is independent of the dialogue input *Q*, the model can learn this part on nondialogue inference datasets. Here, we refer to the previous work [35] and introduce the unlikelihood objective function to reduce the possibility of contradictory data in the inference data so that *D2* can obtain the ability of consistent understanding. We use the pretrained Bert model to initialize all modules. The overall structure of the BoB model and the corresponding training method are shown in Figure 11.

The working principle of the encoder *E* is similar to the standard Bert model, which converts the input text into a word vector through the embedding layer shared by all modules. It then is encoded into a vector representation *H* by the encoder *E* through a multilayer bidirectional self-attention mechanism.

Benefiting from initialization, *D1* inherits Bert's robustness. The difference is that *D1* works as an autoregressive decoder. When Bert predicts a mask word, it uses the left and correct bidirectional information of the word. Still, in the autoregressive generation task, the words are indicated from left to right one by one. In Figure 11, to eliminate this inconsistency, this paper is inspired by UniLM to upper-triangular mask matrices during training and prediction to ensure that the generated reply words can only rely on existing information. In addition, this scheme is similar to the classic sequence-to-sequence model, adding an attention mechanism between the decoder and encoder can effectively improve the performance.

The *D2* is the key for the BoB model to separate understanding and generation tasks. Like *E* and *D1*, *D2* is also initialized from Bert and thus inherits an excellent semantic representation for text understanding tasks. The task of *D2* is to learn how to understand coherence relations and apply this ability to the task of reply generation, so it needs to utilize bidirectional information for better learning. To achieve this, *D2* is trained with both the standard cross-entropy loss for text generation and the unlikelihood loss for consensus understanding.

The BoB model can learn from limited role-based dialogue data by separating reply generation and consistent understanding and introduces the unlikelihood training based on nondialogue reasoning data, which improves the model's consistent understanding ability. The *E*, *D1*, and *D2* are all based on the generic Bert model, and the Bert-base-uncased version can be downloaded directly from the Hugging face [36]. Since the Bert-base-uncased is an already trained model, we only need to train it for task adaptation. Experiments show that the BoB model can achieve better results than strong baseline methods trained with full data, even with less characterized data [34].

## 4. Experiments

This section will experiment with the performance of EDA and the BoB model augmented by EDA. The experiments mainly verify the scheme's performance by comparing the semantic similarity between the original text and the generated text. And this paper especially calculates the similarity of short texts. Generally, it is necessary to embed two short texts and then calculate the cosine similarity. Word embedding methods such as word2vec and GloVe have become standard methods for finding semantic similarities between words. Specifically, it includes the benchmark method based on the average value of word embeddings, the word shift distance method based on the shortest distance of words, the smooth inverse frequency method, and the method based on pretrained encoders [38, 39]. On this basis, many mature short text similarity schemes have been proposed. The pretrained language model (ERNIE) based on Baidu's self-developed performance performs well [40], and it is very convenient to use. The ERNIE model has a more vital semantic understanding ability and can deeply understand the semantic relationship between texts.

Baidu's solution is an online method, where users can send requests to the server in POST mode through the API provided by Baidu. The scheme can return the similarity value of two texts each time, and the maximum length of each text is 512 kB. The *score* represents the similar result, and the value range is (0, 1). The higher the *score*, the higher the similarity.

### 4.1. EDA Performance Analysis

**Figure 12:**
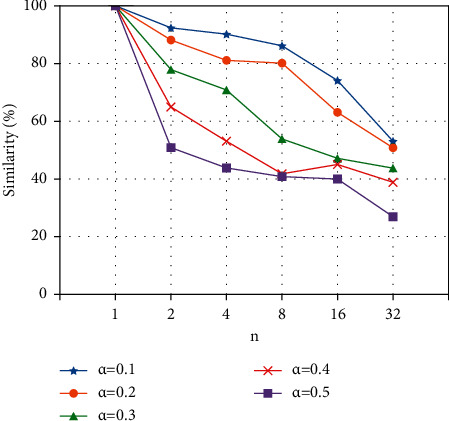
Text semantic similarity with EDA.

In Section 3.1, *n* and *α* are two main parameters of the EDA, where *n* controls the number of target sentences generated corresponding to each source sentence, and *α* is a parameter that indicates the percent of the words in a sentence are changed by augmentation. In our experiment, we modified the value of *n* to {1, 2, 4, 8, 16, 32}, and *α* was {0.1, 0.2, 0.3, 0.4, 0.5}, respectively, and finally analyzed the text semantic similarity between the generated text after EDA augmentation and the original text. For the *n* texts generated from one original text, we take the average semantic similarity of the *n* texts as the comparison value. The experimental results are shown in Figure 12 and Table 1.

In Figure 12, it is first shown that as the value of *n* increases, the text semantic similarity decreases. The reason is that EDA will prioritize adopting a better modification scheme for the original text. When the number of texts generated is larger, the similarity between the generated text and the original text will be lower. Secondly, the increase of the *α* will also increase the degree of modification of the original text by EDA. The reason is that when the *α* increases, more words are changed in the original sentence. However, since EDA only changes the structure and content of the statement by simply replacing, moving, etc., when the *α* is too large, the semantics of the original statement will be changed. This will lead to a large difference in text semantic similarity.

After many experiments, we show that the quality of the generated text is higher when the text semantic similarity is above 75%. For the short text dataset, text augmentation can achieve better results when the combination of *n* and *α* is in the green area of Table 1.

A good evaluation metric can effectively guide the model to fit the data distribution and objectively evaluate the quality of the text generation model. However, due to the inherent complexity of natural language and the limitations of current technology, there is no perfect evaluation indicator yet. Reference [40] lists several types of common [41–44] evaluation indicators: word overlap evaluation metric (WOEM) and word vector evaluation metric (WVEM). The WOEM includes BLEU, ROUGE, and METEOR, and the WOEM is represented by greedy matching, embedding average, and vector extrema. Perplexity (PPL) is a widely used language model-based method because it can also directly compare the pros and cons of two language models in predicting samples. In addition to the abovementioned automatic measurement methods, this paper also introduces several manual measurement methods.

(1) $$\begin{array}{c} PP\left(S\right)={P\left(w_{1}w_{2}\ldots w_{N}\right)}^{-1/N}\\ =\sqrt[{N}]{\frac{1}{P\left(w_{1}w_{2}\ldots w_{N}\right)}}\\ =\sqrt[{N}]{\overset{N}{\underset{i=1}{\prod }}\frac{1}{P\left({w_{i}|w}_{1}w_{2}\ldots w_{i-1}\right)}}. \end{array}$$

For the BoB model, our experimental scheme consists of two parts. The first part is one horizontal comparison scheme. We compare the PPLs of transformer, GPT2, BoB, and EDA-BoB to verify that the EDA-BoB model has high text generation quality. The specific method is to calculate the PPL of each model on the respective test set. And TensorFlow can provide a simple method for calculating PPL. Specifically, when using the *tf.contrib.seq2seq.sequence_loss()* function to calculate the *loss* of the model, take the exponential operation on the *loss* directly. The results are shown in Table 2. Compared with the first two models, BoB has apparent advantages, and EDA can improve BoB.

**Figure 13:**
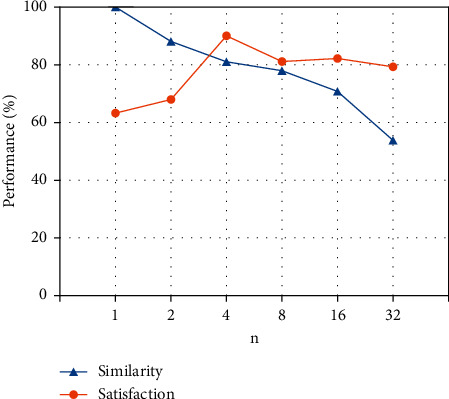
EDA-BoB performance.

On the other hand, we adopted a similar scheme to the EDA experiment. We set the *α* to 0.2 and analyze the similarity between the text generated by our model and the text in the test set when the *n* = {1, 2, 4, 8, 16, 32}. Meanwhile, we also conducted a human survey to determine the augmentation effect of EDA on BoB. The scheme is based on human subjective satisfaction with the generated text, which can better reflect the richness and consistency of the text. Thirty volunteers from the computer science department of the author's institution each obtained 20 sets of data, each consisting of a question and an answer. And volunteers were asked to judge the quality of the text on a scale of 1 to 100. The higher the ranking, the higher the satisfaction. The experiment also analyzed the change in volunteer satisfaction when the *n* = {1, 2, 4, 8, 16, 32}, and the results are shown in Figure 13. The volunteers are generally satisfied with the quality of the text generated by EDA-BoB, and the increase of the *n* value has a slight improvement to the model.

But we can also see from the results that when the *n* is increasing, the similarity between the generated text pair and the original text pair is always decreasing. The main reason is that the more text pairs are generated, the more different the model is from the generated results, and the lower the match between the question and the answer. Meanwhile, the satisfaction reaches the highest 92% when *n* is 4, and then declines and remains at 80%. This shows that the increase of the *n* has an obvious effect on the quality improvement of the generated text in the early stage, but too much EDA augmented data will not continue to improve the model.

## 5. Conclusion

The limited size of the training corpus and the lack of emotion in the generated sentences are the two major problems for text generation tasks. Based on text augmentation methods and knowledge understanding mechanisms, this paper proposes a lightweight language model (EDA-BoB). This model uses a lightweight text augmentation approach (EDA) to expand the training dataset size, which can improve model performance and reduce overfitting. Furthermore, this paper adopts a multi-Bert model (BoB) based on the understanding mechanisms for text generation. And this BoB model can learn user role information and use it to generate text with richness and consistency. Experiments show that EDA can further improve the performance of BoB with a small computational cost. Our future work will focus on researching lightweight text augmentation methods for large datasets and designing general solutions for multiple language models.

## Figures and Tables

**Table 1:** Text semantic similarity with EDA (1).

***α***	***n***					
	***n*** **=** **1**	***n*** **=** **2**	***n*** **=** **4**	***n*** **=** **8**	***n*** **=** **16**	***n*** **=** **32**
***α*** **=** **0.1**	100	92	90	86	75	56
***α*** **=** **0.2**	100	88	81	80	63	51
***α*** **=** **0.3**	100	78	71	54	47	44
***α*** **=** **0.4**	100	65	53	43	45	39
***α*** **=** **0.5**	100	50	44	42	40	27

BoB performance analysis.

**Table 2:** Text semantic similarity with EDA (2).

Model	PPL
Transformer	28.8
GPT2	14.4
BoB	7.73
EDA-BoB	7.35

## Data Availability

The datasets used and analyzed during the current study are available from the corresponding author upon reasonable request.
